# Male Agonistic Behavior on Atlantic Cod Spawning Grounds

**DOI:** 10.1002/ece3.73316

**Published:** 2026-03-27

**Authors:** J. E. Skjæraasen, P. Arechavala‐Lopez, Ø. Karlsen, E. M. Olsen, J. J. Meager, D. Nyqvist, L. S. Doksæter, K. McQueen, K. de Jong, K. H. Rugtveit, I. A. Catalan, Ø. Langangen

**Affiliations:** ^1^ Institute of Marine Research Bergen Norway; ^2^ Mediterranean Institute for Advanced Studies (IMEDEA, CSIC‐UIB) Esporles Spain; ^3^ Institute of Marine Research Flødevigen Marine Research Station His Norway; ^4^ Centre for Coastal Research (CCR), Department of Natural Sciences University of Agder Kristiansand Norway; ^5^ Natural Resources, GHD Maroochydore Queensland Australia; ^6^ Department of Aquatic Resources, Institute of Freshwater Research Swedish University of Agricultural Sciences Drottningholm Sweden; ^7^ Department of Biosciences University of Oslo Oslo Norway

**Keywords:** accelerometer, agonistic interactions, gadoids, *Gadus morhua*, leks, mating system, sexual selection, spawning, telemetry

## Abstract

The mating system of the iconic Atlantic cod ( *Gadus morhua* ) has been described as a lek, a mating system where male–male agonistic interactions are expected. However, observing such behaviors directly in marine environments is challenging. So far, the evidence supporting the importance of agonistic interactions between males is mainly derived from laboratory studies with fish kept in close confinement, possibly elevating the observed levels and their perceived importance. In natural aquatic systems, agonistic interactions may be inferred from acoustic telemetry transmitters fitted with accelerometers, given that they are associated with high activity levels. A large telemetry dataset on Atlantic cod was collected during (i) a field study at two Western Norway spawning grounds from 2019 to 2024 and (ii) a close confinement net—pen study in 2019–2020. A total of 154 sexed mature Atlantic cod were tagged with acoustic transmitters fitted with accelerometers and depth sensors, yielding ~1.5 million accelerometer detections for subsequent activity analyses. In the net‐pens, male cod showed much higher activity levels than females. Overall activity levels were reduced in the field, but male activity levels remained higher than those of females during the spawning period. A prominent feature of coastal cod spawning along the Norwegian coast is that males tend to dwell deeper than females. We therefore explored whether depth influenced activity patterns of males and females. This was indeed the case with males being more active than females at fish depths > 20 m and no sex‐difference in activity levels closer to the surface (< 20 m depth). No sex difference in activity levels was found outside the spawning period. Our data suggest that male–male agonistic interactions, inferred from activity levels, are present on natural spawning grounds for Atlantic cod, albeit at reduced levels compared to levels exhibited in net–pen confinement. We hypothesize that the heightened activity levels of males compared to females when occupying deeper waters are related to their overall deeper distribution and territorial and agonistic behavior at these depths, agreeing with the lek description of the cod mating system.

## Graphical abstract

The mating system of the Atlantic cod ( *Gadus morhua* ) has been described as a lek, a mating system where male–male aggressions are expected. However, observing aggressions directly in marine environments is challenging. Using accelerometer tags we show that male–male agonistic interactions, inferred from activity levels, are indeed present on natural spawning grounds for Atlantic cod, albeit at reduced levels compared to levels exhibited in net‐pen confinement.

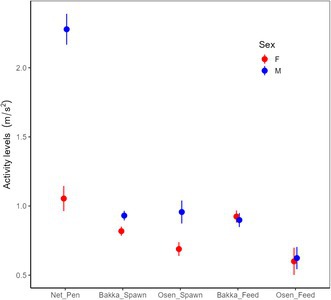

## 1 Introduction

Teleost fish are the most numerous vertebrate group on Earth, with approximately 30,000 species identified to date (Helfman et al. 2009). This group exhibits remarkable reproductive diversity (Smith and Wootton 2016). The most common teleost reproductive mode is broadcast spawning, where fish release their gametes into the water column. A notable group of broadcast spawners is the gadoids, a group of mainly demersal fish predominantly residing in the Northern Atlantic (Howes 1991). The most commercially and culturally important gadoid is the Atlantic cod ( *Gadus morhua* ), which has supported fisheries and human settlements on both sides of the Atlantic for millennia (Hutchings and Rangeley 2011; Martinez‐Garcia et al. 2022). Once described as a promiscuous, broadcast group spawner where sexual selection is primarily driven by sperm competition (Stockley et al. 1997) and where there is little opportunity for mate choice (Berglund 1997; Morgan et al. 1999), this description has since been refuted by a large body of evidence. Laboratory studies have demonstrated courtship behavior (Table S1), for example, males following and circling females in their vicinity and engaging in “ventral mounts” or a mating embrace, whereby the male is positioned underneath the female, gonadal pores close together, clasping her with his enlarged pelvic fins inducing gamete release by both sexes (Brawn 1961b; Hutchings et al. 1999; Skjæraasen, Meager, and Hutchings 2010). Male—male agonistic interactions (Table S1), such as chasing, involving rapid swimming toward another male, prodding, or other rapid approaches toward other males is also frequently observed in confinement (Brawn 1961b; Hutchings et al. 1999; Skjæraasen, Meager, and Hutchings 2010). Sound production is also associated with these behaviors and commonly heard during spawning (Hawkins and Rasmussen 1978; Rowe and Hutchings 2006; Skjæraasen et al. 2012). These mating behaviors are suggested to form the basis for female choice (Skjæraasen, Meager, and Hutchings 2010; Skjæraasen, Meager, Karlsen, et al. 2010), resulting in large male reproductive skews with some males siring a disproportionate amount of eggs (Bekkevold 2006; Rowe et al. 2008). Field observations have further identified male aggregations at spawning grounds, which females apparently approach when ready to spawn (Morgan and Trippel 1996). Individual females, particularly the larger ones, will also often visit multiple spawning sites during one season (Olsen et al. 2023).

The cod mating system is, therefore, currently more often described as a lek (Nordeide and Folstad 2000; Windle and Rose 2007). In lekking species, male—male agonistic behavior is common and often related to competition for small territories (Rathore et al. 2023). The outcome of such interactions may impact male reproductive success by allowing males to obtain central positions favored by females (Bro‐Jorgensen 2002; Shorey 2002; Small et al. 2009) or through female preference for large, dominant males, because, as has been suggested for cod (Rowe et al. 2008) and bumphead parrotfish *Bolbometopon muricatum* (Munoz et al. 2014), this may minimize the chance of interrupted matings. However, observing agonistic interactions directly in the aquatic realm is challenging, which is particularly true for cod, given that spawning typically occurs at depths > 30 m (Meager et al. 2009; Skjæraasen et al. 2024). The evidence supporting the importance of male–male agonistic interactions for the cod mating system is thus hitherto derived from laboratory studies where cod have been kept in close confinement. Interestingly, Dean et al. (2014) noted that male cod on a Massachusetts spawning ground generally had non‐overlapping territories. They suggested that the levels of agonistic interactions seen in the laboratory may be elevated (i.e., biased) due to the higher densities or increased encounter rates with confinement also preventing subordinates from avoiding conflict through dispersal (Sloman and Armstrong 2002), although the relation between density and agonistic behavior is not necessarily linear (Fenderson and Carpenter 1971; Weir et al. 2011; de Jong et al. 2012).

One indirect way of detecting fish activity levels in the oceanic environment is the use of telemetry techniques, and, specifically, the use of accelerometer acoustic transmitters. These transmitters typically record movement along the *X*, *Y*, and *Z* axes during listening windows, which are transmitted as one averaged value and detected by receivers deployed in the water column (Lennox et al. 2023). High‐energy behaviors, such as predatory feeding events and agonistic interactions (e.g., male–male interactions during cod spawning), typically involve short bursts of intense activity, including chases that elicit fleeing responses in the recipient (Brawn 1961a; Hutchings et al. 1999). These behaviors can therefore be detected as high values in the accelerometer data (Nakamura et al. 2011; Watanabe et al. 2019). Here, we take advantage of this methodology to examine the putative occurrence and importance of male—male agonistic interactions for naturally spawning Atlantic cod. First, we analyze sex‐specific activity levels in a confined net‐pen study and compare them with activity recorded at two natural spawning grounds in western Norway during spawning. We then assess how these patterns differ between the spawning season and the non‐spawning (feeding) period.

## 2 Methods

### 2.1 Fish Capture and Tagging

**FIGURE 1:**
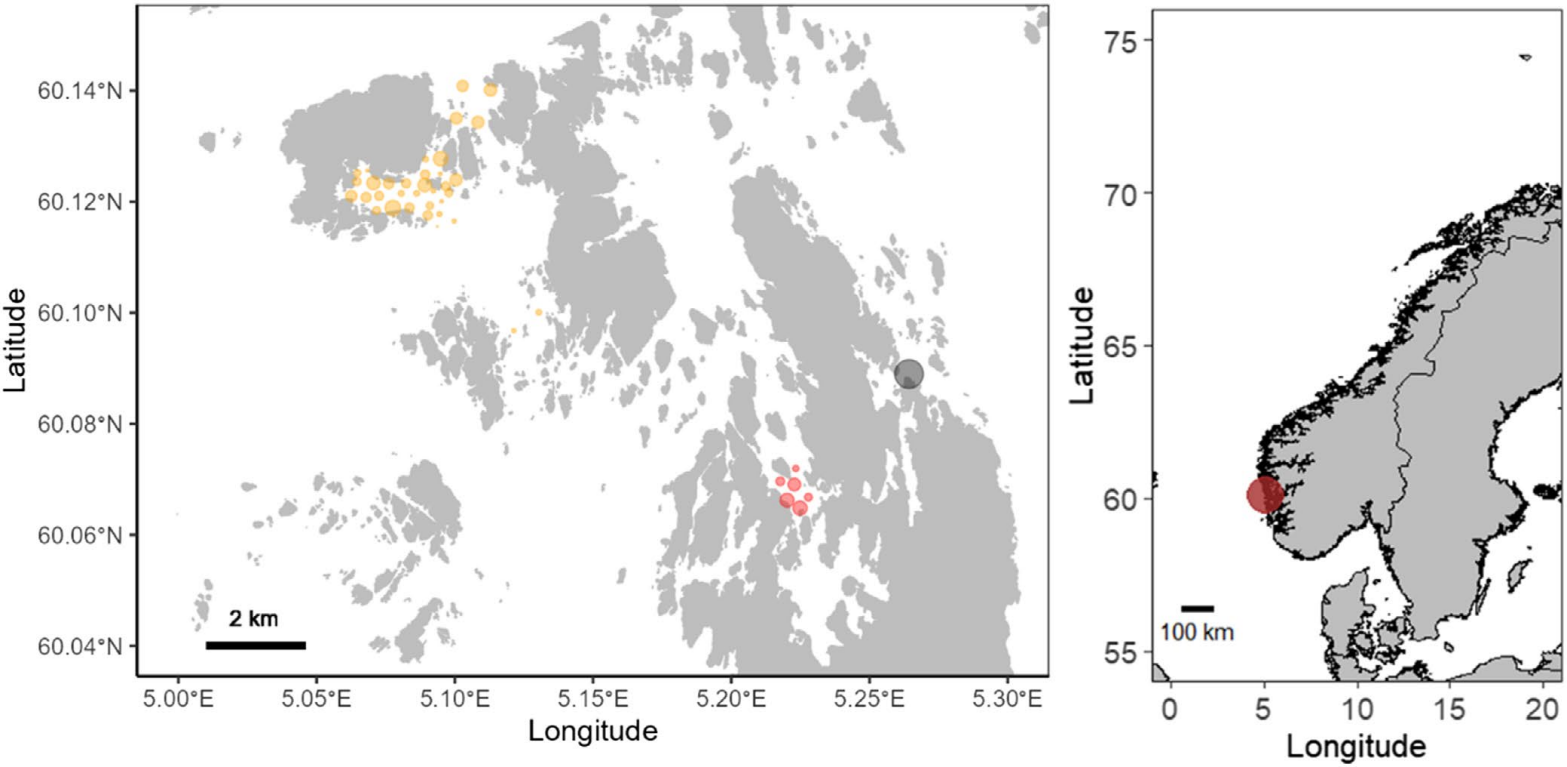
Map of the study area. Left panel: the Bakkasund spawning ground grid (yellow points), Osen spawning ground grid (red points), and the site of the net‐pen study (black point). For Bakkasund and Osen, the size of the points is scaled to the number of detections at each logger. Right panel: location of the study area on the Norwegian coast.

Cod were caught in December or January 2019–2024 using baited pots and gillnets. The fish were kept in net‐pens for 1–2 weeks within the two study areas of Bakkasund and Osen, until tagging and release, or transfer to net‐pens. Prior to tagging, fish were anesthetized in a bath of seawater and MS‐222 (50 mg/L seawater). Subsequently, each cod was measured for weight and length, and sexed using ultrasound (Mindray DP‐50Vet equipped with a 75L50EAV 5–10 MHz transducer (Shenzhen Mindray Bio‐Medical electronics, China), where sex was determined by visual appearance of gonads and maturity by gonad diameter as described by Karlsen and Holm (1994)). Egg biopsies were obtained from all females with subsequent analysis of oocytes by image analysis, allowing confirmation of sex and spawning readiness (Thorsen and Kjesbu 2001). An acoustic tag was inserted in the body cavity through an incision made on the ventral side of the fish, which was then closed by two sutures. Two types of acoustic tags transmitting accelerometer data have been used during the study (V13AP, V13TP ADST tags, Innovasea, Canada). These tags transmit unique identity codes (IDs) at 69 kHz. Transmissions occurred at random intervals, on average every 250 s with a minimum and maximum delay of 200 and 300 s, respectively, except in 2024 when the minimum and maximum delays were set to 40 and 60 s. Tags with more than one sensor alternated transmissions between the sensors (1:1 ratio). The accelerometer data is transmitted from the tags (Tables 1 and 2) as one single smoothed value derived from a 25 s period that samples movement in the *X*, *Y*, and *Z* directions at a frequency of 12.5 Hz. The minimum and maximum value transmitted was 0 and 3.43 m/s^2^, respectively, except for accelerometer tags used in 2024, where the minimum and maximum values transmitted were 0 and 4.9 m/s^2^. In addition to the internal tagging, an external T‐bar tag (TBA standard anchor t‐bar tag; Hallprint, Australia) was anchored at the base of the anterior dorsal fin for visual recognition of tagged fish. After tagging, cod were returned to a tank filled with a constant supply of seawater to recover from the tagging procedure, before being released at central positions within their respective spawning grounds of capture, or into the net‐pens (Figure 1). Here, we only consider data from sexually mature cod tagged with accelerometer tags for further analyses (n_total_ = 154, n_field_ = 138, n_netpen_ = 16, Tables 1 and 2). The study was approved by the Norwegian Food Safety Authority, permission nos. 18,034 (2019–2021), 26,019 (2021), 28,733 (2022–2023), and 29,943 (2023–2024).

**TABLE 1:** Summary data for the net‐pen study. “Net‐pen” denotes different net‐pens. Fish is the total number of fish in each net pen at the experimental start, and Tagged fish is the number of cod tagged with accelerometer tags. The numbers of males (M) and females (F) in the respective net‐pens are given in parentheses. Mean length is the average length of the tagged fish.

Year	Net‐pen	Fish (n)	Tagged fish (n)	Mean length (cm)
2019	1	17 (10 F, 7 M)	1 (1 F)	62 (F)
2019	2	15 (9 F, 6 M)	2 (1 M, 1 F)	68 (F), 59 (M)
2019	3	17 (10 F, 7 M)	1 (1 M)	52.5 (M)
2020	1	20 (11 F, 9 M)	4 (2 F, 2 M)	78.5 (F), 63 (M)
2020	2	20 (10 F, 10 M)	4 (2 F, 2 M)	67 (F), 54 (M)
2020	3	17 (9 F, 8 M)	4 (2 F, 2 M)	72 (F), 70 (M)

**TABLE 2:** Summary data for the cod tagged with accelerometer tags in Bakkasund and Osen yearly from 2019 to 2024 that yielded data for the present study. F represents females, M represents males, n is the number of fish tagged, and L is their mean length.

Year	Bakkasund				Osen			
	F (n)	FL (cm)	M (n)	ML (cm)	F (n)	FL (cm)	M (n)	ML (cm)
2019	3	73.4	4	57.3				
2020	11	70	10	62.4	7	53.7	3	51.8
2021	4	59.1	9	49.5	7	53.4	8	52.4
2022	6	68.3	14	61.8				
2023	4	70.0	7	54.4				
2024	21	60.6	20	56.4				

### 2.2 Net‐Pen Study

A net‐pen study was conducted at IMR's research facility at Austevoll, western Norway (Figure 1). Here, standard aquaculture nets with an internal circular bag, both nets made of tarpaulin, were mounted on a square net frame at the surface. The circular bag housing the cod (*n* = 15–20, Table 1) was 5 m in diameter and 5 m deep with an additional 1‐m deep central cone (Figure S1). All net‐pens received a continuous flow of deep water supplied at the surface at a rate of 100 L/min. The fish were kept in the pens from March 22 to April 15, 2019, and from Feb 28 to March 19, 2020. Monitoring of spawning behavior during this time was made possible through side‐mounted underwater video cameras. Over the 2 years, 16 fish were tagged with acoustic telemetry tags (Table 1) following the procedure described above. Tag transmissions were detected by VR2W receivers placed just outside the net‐pen area. During the spawning period, fish were exposed to short intermittent noise from a downscaled seismic air‐gun. Although no noise impact on acceleration values was detected (Rugetveit 2024), the period where fish were exposed to noise (Table S2) was omitted from the dataset used for final analyses. This yielded a final dataset of 20,997 accelerometer detections for analyses from February and March.

### 2.3 Field Study

The field study was conducted at two known cod spawning grounds near Austevoll: Bakkasund and Osen (Figure 1). These two spawning grounds have comparable maximum depths of about 80–100 m. The tagged cod (Table 2) were monitored by fixed arrays of acoustic telemetry receivers (models VR2Tx and VR2AR, Innovasea, Canada, Figure 1) that were deployed on each spawning ground in November 2018. These grids have enabled studies on general cod behavior and movement on both grounds since then (e.g., Skjæraasen et al. 2024). The Bakkasund grid is still operational, whereas the Osen grid was discontinued and removed in June 2023. Receiver maintenance and data download have been conducted at least once per year during early summer (May–June) after the spawning season. Since 2020, the Bakkasund spawning ground has also been used intermittently as a test site for investigating behavioral responses of fish to anthropogenic sound sources. Cod tagged at the Bakkasund spawning ground were therefore exposed to anthropogenic sound (seismic survey sources, ship noise, and playback of low‐frequency sound) during controlled exposure experiments in the spawning seasons of 2020, 2021, 2022, and 2024, and the feeding period of 2023. Each exposure experiment lasted only between 3 and 5 days, with fish being exposed to sounds intermittently during these periods. Similar to the net‐pen study, any behavioral changes, if detected at all, were typically minor and/or transient (McQueen et al. 2022, 2023, 2025); however, in line with the treatment of the net‐pen study, data from all exposure periods were omitted from final analyses (Table S2). For further details on grid deployment, fish capture, and the study sites we refer to McQueen et al. (2022); McQueen et al. (2023); McQueen et al. (2024).

### 2.4 Data Filtering, Preparation, and Analyses

All analyses were performed using R version 4.3.0 (R Core Team 2023). The base library of R and the *tidyverse* packages (Wickham et al. 2019) were used for data organizing.

#### 2.4.1 Initial Data Filtering and Data Preparation of Field Data

To correct for receiver clock drift, a linear correction based on the satellite clock time stamp at receiver initiation and download was first applied, using the autocorrect function in the software provided by the receiver manufacturer (VUE, Innovasea). The time‐corrected datasets were used for all further data analyses within R and span the period from January 29, 2019 (first release of fish) to June 7, 2024 (last data download relevant for the present study).

Prior to statistical analyses, the time‐corrected field data were filtered to (i) remove fish that were detected but did not exhibit vertical movement for > 1 day and, thus, were assumed to be dead (Villegas‐Ríos et al. 2020), (ii) remove repeated counts of the same signal transmission detected at several stations, and (iii) remove dubious/erroneous detections, defined as a single daily detection of a given fish ID within the grid. For (i), all data were removed if the fish never displayed any vertical movement, whereas only the period after vertical movement ceased was removed if fish initially displayed vertical movement. For (ii), we only included the first recorded detection if any data with the same fish ID and sensor value were detected within the minimum tag delay at multiple stations.

The tags alternate between transmitting depth and accelerometer values. To be able to employ fish depth as an explanatory variable in data analyses of the field data (see below), we assigned each accelerometer transmission to the depth detection closest in time for the same fish. If no depth detections were found within 1800 s before or after the accelerometer transmission, the data point was removed from the final dataset used for analyses. Overwhelmingly, the depth transmission before or after the accel transmission was used to assign the accelerometer transmission depth; that is, the depth assigned to the accelerometer detections in 2019–2023 had typically been observed 200–300 s before or after the accelerometer detection. This procedure resulted in the assignment of 98% of the accelerometer transmissions to a depth. The analyses were further restricted to fish depths ≤ 60 m due to few detections (< 2%) deeper than this, and only fish data from the tagging year were included. The tags used in 2024 transmit data ~5 times more frequently than in the other tagging years. To minimize effects on transmission frequency on our results, data for the 2024 fish were trimmed, and only every fifth activity measurement for each fish was included in the final analysis. This yielded final datasets of 752,102 and 272,059 accelerometer transmissions from the wider Bakkasund spawning ground area and the Osen spawning ground, respectively (Figure 1).

### 2.5 Data Analyses

#### 2.5.1 Net‐Pen Data

The accelerometers transmit right‐censored data, that is, if the recorded smoothed value is at or above the threshold value, they deliver the threshold (maximum) value. This right censoring was particularly obvious in the net‐pen data (Figure S2). To deal with this censoring, we used data imputation for the threshold values for both the net‐pen and field data using the *censlm* package (Marttila 2024). For further details on this procedure, see the Supporting Information. The imputed data (Figure S2) were then aggregated to deal with zero inflation at the level of individual fish. For each fish, the mean of all acceleration values recorded during the study period was used as the dependent variable in the analyses; that is, each fish contributed one data point to the analysis. The categorical variable sex and the continuous variable fish length, and their interaction were included as explanatory variables in the initial starting model using base R. Aggregated values for individual fish were given a weight equal to the log of the number of accelerometer detections to account for variable detection numbers. Model selection was performed by use of the ‘dredge’ command of the *MuMIn* package (Barton 2020) to arrive at the most parsimonious model with the lowest AICc score. All variables included in the best model or in a model within 2 AICc units of the best model were retained for the final model used in analyses (Hurvich and Tsai 1989). Final model validation was performed by visual inspection of the residuals. This model selection and model validation procedure was also used for the models on the field data described below.

#### 2.5.2 Field Data

We first split the field data into two different seasons: a spawning period lasting from February to March and a feeding period lasting from May to December, thus omitting the transitional months of January and April. We first looked at overall sex differences in activity levels in Osen and Bakkasund. For these analyses, we used the same aggregation method for the activity response variable as described above for the net‐pen data and again weighted the aggregated mean values for individual fish by the log of the number of detections. In Osen, we used the same linear starting model as employed for the net‐pen data. For Bakkasund, we employed a mixed‐effect model using the lmer function of the lme4 package in R (Bates et al. 2015) with activity level as the response variable and the categorical variable sex, the continuous variable length, and their interaction as fixed effects with year, given that we had 6 years of data for Bakkasund, also included as a random effect. The same starting models for Osen and Bakkasund were employed separately for the spawning period and, in contrast, the feeding period.

**FIGURE 2:**
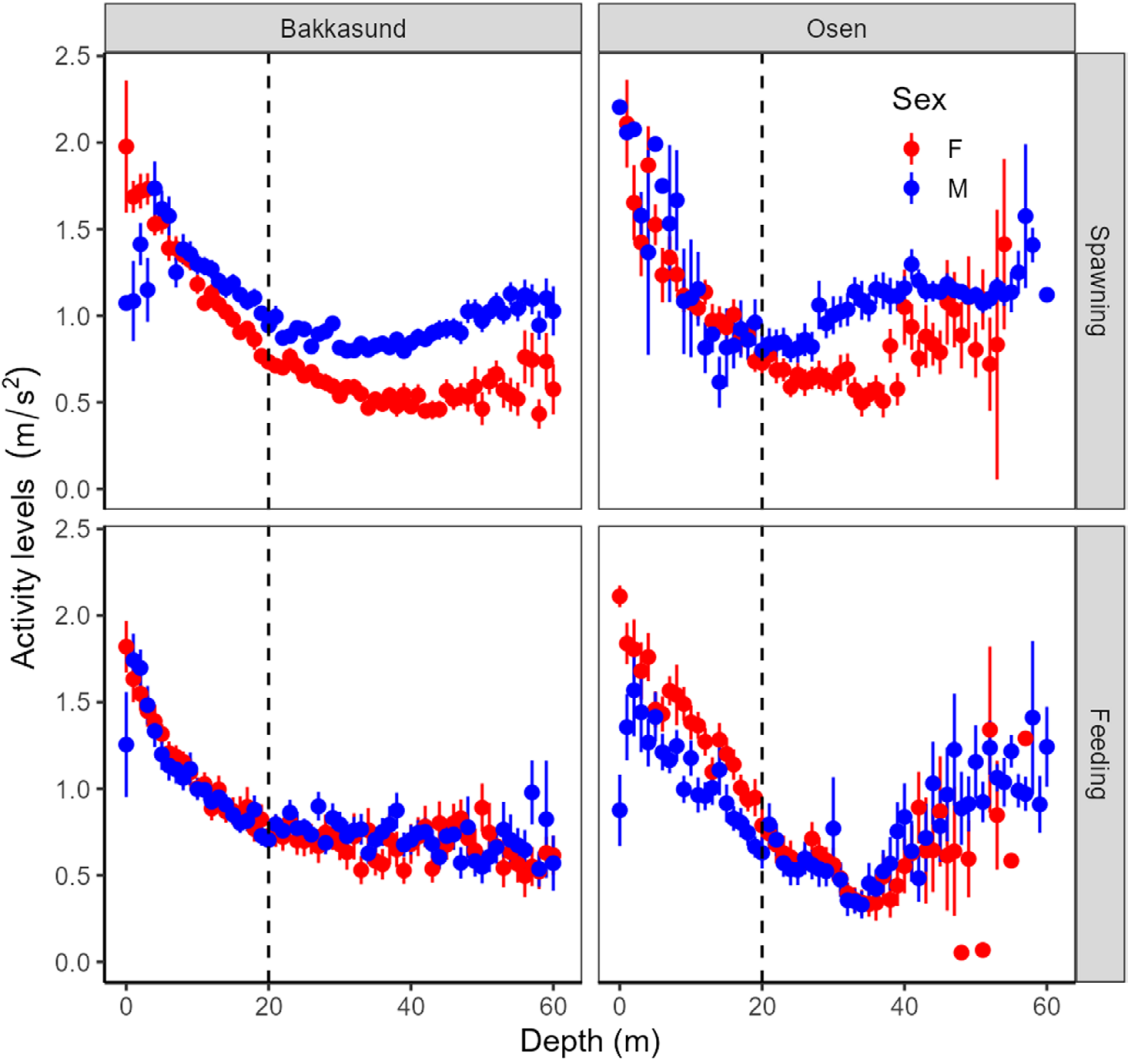
Summary exploratory plot showing overall activity levels for males and females for every 1 m from the surface to 60 m depth for males and females for Bakkasund and Osen. Mean values were first calculated for every individual for all depth bins. The average of these mean values ±1 SE is shown in the plot.

Male cod typically stay deeper than females during the spawning period along the Norwegian coast (Meager et al. 2009; Barth et al. 2019; Skjæraasen et al. 2024). It was therefore hypothesized that activity levels differ with depth between the sexes. Indeed, exploratory plots indicated clear sex‐dependent patterns of accelerometer values with depth during the spawning period, but not during the feeding period (Figure 2). To examine these aspects more thoroughly, more complex models were run for the spawning period only at both study grounds. These latter models included the categorical variables sex and depth category (< 20 m, > 20 m) and their interaction as explanatory variables in the starting model, with FishID and year (Bakkasund only) treated as random effects. Data were again aggregated at the level of individual fish, and the mean activity level for individual fish was used as the response variable. However, given that the same fish typically was detected in both deep and shallow water layers and hence yielded two values for the analysis, FishID was incorporated as a random effect. The *glmmTMB* package (Brooks et al. 2017) employing a gamma distribution with a log‐link function was used for model fitting. Activity levels for individual fish were again weighted by the log of the number of detections.

The analyses described above provide information on overall activity levels for males and females and how it interacts with fish depth. We also wanted to explore if the proportion of activity measurements likely associated with high activity and agonistic interactions, such as chasing and burst movements, differed between sexes and how it might interact with fish depth during the spawning period. Previous studies on wild cod, where acoustic transmitters equipped with accelerometers were used to assess their daily patterns or behavioral states, found that acceleration values higher than 2 m/s^2^ are relatively uncommon (van der Knaap et al. 2021; McQueen et al. 2024). Similarly, studies in laboratory swim trials have reported that faster movements of cod (burst, sprint) show the highest acceleration values (above 2–3 m/s^2^) (Videler 1981; Nelson et al. 2002; Broell et al. 2016). We, therefore, denoted activity levels > 2 m/s as high activity and calculated the proportion of high activity levels in shallow (< 20 m) and deep water (> 20 m) for each fish. This value (P_HV) was then employed as the dependent variable in beta regression models. We used beta regressions as these produce less biased estimates and more straightforward statistical inference when dealing with continuous proportions (Douma and Weedon 2019). We used the *glmmTMB* command with the beta‐family and logit link function from the *glmmTMB* package for model running (Brooks et al. 2017). As beta regression models only allow proportions in the range (0,1), the data were first transformed following equation (1) in Douma and Weedon (2019). The categorical variables sex and depth category (< 20 m, > 20 m) and their interaction were incorporated as explanatory fixed effect variables in the starting model, with FishID and year (Bakkasund only) treated as random effects. Transformed P_HV values for individual fish were again weighted by the log of the number of detections.

## 3 Results

### 3.1 Net‐Pen Study

**FIGURE 3:**
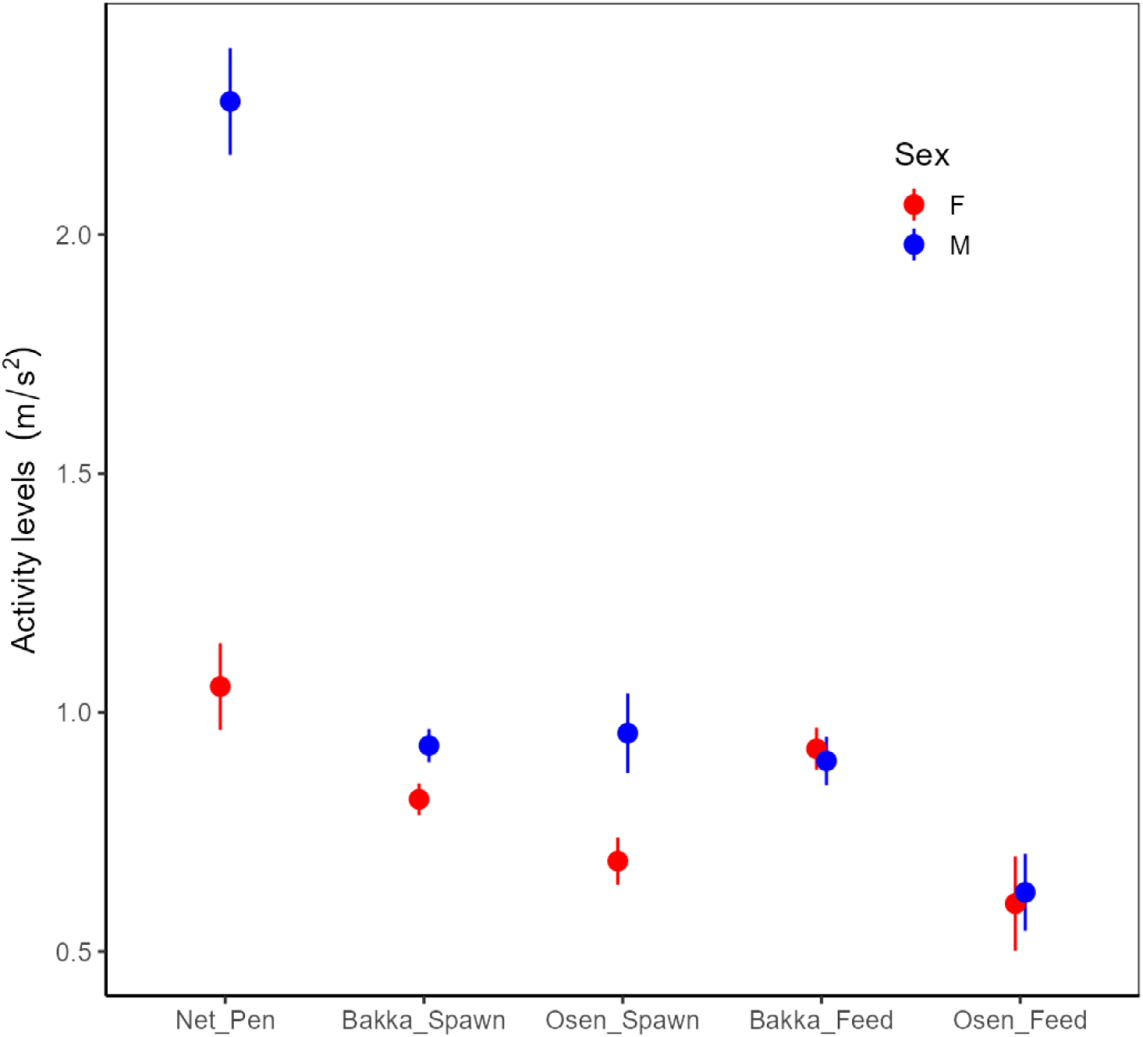
Summary plot of the activity levels for males and females in the net‐pen study and the field. Mean values were first calculated for every individual. The average of these mean values ±1 SE is shown in the plot.

Males showed very high activity levels and significantly more so than females (*p* < 0.0001, Table 3 and Figure 3) in the net‐pen. A slight negative effect of fish length was also retained in the final model (Table 3).

**TABLE 3:** Summary of the activity level results for the net‐pen study. The treatment contrast of R was used in the analyses, with the intercept value depicting the value for females. Brackets indicate the categorical variables sex, that is, males (M), tested against this reference value.

Predictors (Intercept)	Activity levels		
	Estimates	CI	*p*
	1.82	0.70–2.93	**0.004**
Sex [M]	1.11	0.78–1.45	**< 0.001**
Length	−0.00	−0.00–0.00	0.155
Observations	16		
*R* ^2^/*R* ^2^ adjusted	0.860/0.839		

*Note:* Significant *p* ‐ values (*p* < 0.05) are given in bold.

### 3.2 Field Study

#### 3.2.1 General Model

The overall activity levels, especially for males, were lower in the field compared to in the net‐pen (Figure 3). Even so, male values were higher than female values at both spawning grounds during the spawning period (Figure 3 and Table 4). In contrast, there was no sex difference in activity levels during the feeding period (Figure 3) (Table S3). Body length was selected as an explanatory variable in the final model for the feeding period but not for the spawning period in Osen and not for any periods in Bakkasund (Table 4 and Table S3).

**TABLE 4:** Summary of the activity level results for the general models for Bakkasund and Osen during the spawning period. The treatment contrast of R was used in the analyses, with the intercept value depicting the value for females. Brackets indicate the categorical variable sex, that is, males (M), tested against this reference value. The top part of the model shows the results for the fixed effects, and the bottom part shows the random effects.

Predictors (Intercept)	Activity levels Bakkasund			Activity levels Osen		
	Estimates	CI	*p*	Estimates	CI	*p*
	0.82	0.71–0.92	**< 0.001**	0.70	0.59–0.81	**< 0.001**
Sex [M]	0.11	0.02–0.20	**0.014**	0.31	0.13–0.48	**0.001**
Random effects						
σ^2^	0.39					
τ_00_	0.01 year					
ICC	0.02					
N	6 year					
Observations	112			28		
Marginal R^2^/Conditional R^2^	0.008/0.032			0.337/0.311		

*Note:* Significant *p* ‐ values (*p* < 0.05) are given in bold.

#### 3.2.2 Depth‐Dependent Patterns

**FIGURE 4:**
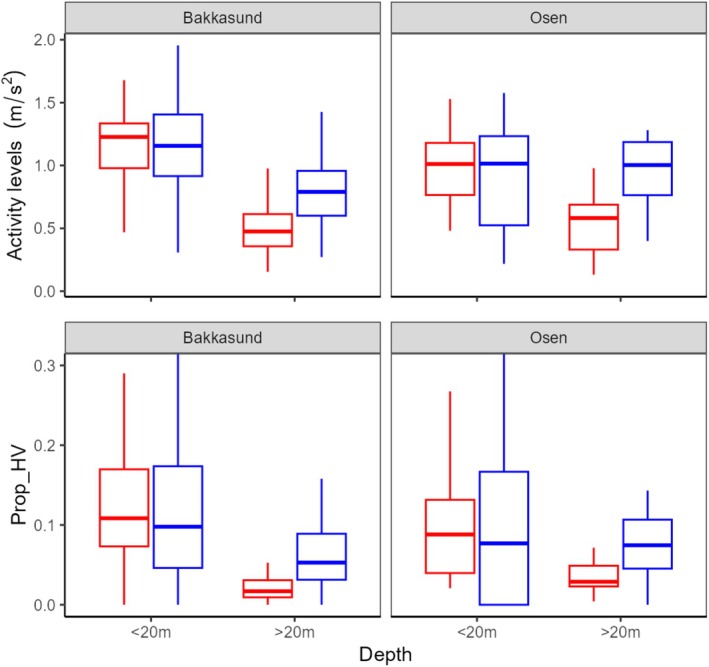
Summary boxplots of the activity levels and proportion of high values (Prop_HV). The proportion of activity levels > 2 m/s^2^, for males (blue) and females (red) in shallow (< 20 m) and deep water (> 20 m) at the Bakkasund and Osen spawning grounds.

The more complex models for Bakkasund showed higher overall activity levels for both males and females in the upper water column (less than 20 m from the surface) than when in deeper waters, with no overall sex difference in activity levels in the upper water column (Figure 4 and Table 5). In deeper waters, males had significantly higher activity levels than females (*p* < 0.0001, Table 5 and Figure 4). In Osen, there was again no difference between male and female activity levels in the upper water column. In deeper waters, female activity levels were strongly reduced compared to the upper water column. This was not the case for males, resulting in significantly higher levels for males compared to females here (*p* < 0.0001, Table 5 and Figure 4). The proportion of high activity measurements (P_HV) showed the same pattern as the activity levels, that is, there was no difference in shallow water, but a significantly higher proportion of high activity values for males in deeper waters (*p*s < 0.0001, Table 6).

**TABLE 5:** Summary of the activity level results for the more complex models for Bakkasund and Osen during the spawning period. The treatment contrast of R was used in the analyses, with the intercept value depicting the value for females in shallow water less than 20 m. Brackets indicate the categorical variables sex, that is, males (M), or depth category, that is, deeper waters (> 20 m), and their interaction tested against this reference value. The top part of the model shows the results for the fixed effects, and the bottom part shows the random effects.

Predictors	Activity levels—Bakkasund			Activity levels—Osen		
	Estimates	CI	*p*	Estimates	CI	*p*
(Intercept)	0.16	0.05–0.28	**0.005**	0.01	−0.16 – 0.18	0.903
Sex [M]	−0.04	−0.18 to 0.10	0.615	−0.17	−0.44–0.09	0.196
Depth Cat [ > 20 m]	−0.97	−1.00 to −0.93	**< 0.001**	−0.73	−0.81 to −0.65	**< 0.001**
Sex [M] × Depth Cat [> 20 m]	0.55	0.50–0.60	**< 0.001**	0.83	0.70–0.97	**< 0.001**
Random effects						
σ^2^	0.05			0.07		
τ_00_	0.13 Serial			0.10 Serial		
	0.00 year					
ICC	0.73			0.59		
N	112 Serial			28 Serial		
	6 year					
Observations	212			54		
Marginal R^2^/Conditional R^2^	0.436/0.843			0.350/0.732		

*Note:* Significant *p* ‐ values (*p* < 0.05) are given in bold.

**TABLE 6:** Summary of the results for the proportion of high accel values (P_HV) for Bakkasund and Osen during the spawning period. The treatment contrast of R was used in the analyses, with the intercept value depicting the value for females in shallow water less than 20 m. Brackets indicate the categorical variables sex, that is, males (M), or depth category, that is, deeper waters (> 20 m), and their interaction tested against this reference value. The top part of the model shows the results for the fixed effects, and the bottom part shows the random effects.

Predictors (Intercept)	P_HV Bakkasund			P_ HV Osen		
	Estimates	CI	*p*	Estimates	CI	*p*
	−2.03	−2.24 to −1.83	**< 0.001**	−2.13	−2.46 to −1.79	**< 0.001**
Sex [M]	−0.04	−0.31 to 0.23	0.761	−0.22	−0.74 to 0.30	0.400
Depth Cat [> 20 m]	−1.60	−1.70 to −1.50	**< 0.001**	−1.06	−1.20 to −0.92	**< 0.001**
Sex [M] × Depth Cat [> 20 m]	0.81	0.68–0.93	**< 0.001**	0.82	0.59–1.06	**< 0.001**
Random effects						
σ^2^	0.21			0.17		
τ_00_	0.44 Serial			0.43 Serial		
	0.00 year					
ICC				0.72		
N	112 Serial			28 Serial		
	6 year					
Observations	212			54		
Marginal R^2^/Conditional R^2^	0.661/NA			0.229/0.779		

## 4 Discussion

Using acoustic telemetry accelerometer tags, we investigated activity levels of male and female cod in a confined net‐pen study and contrasted these to activity levels at two spawning grounds. Males confined to net‐pens were much more active than females, whereas this difference between sexes was less pronounced on natural spawning grounds. Importantly, males were more active than females during the spawning season, irrespective of whether they were in net‐pens or on spawning grounds. In contrast, there was no sex difference in activity levels outside the spawning period. The sex difference in activity levels during spawning further manifested itself as elevated values for males compared to females when in deeper waters, while no sex differences were observed in shallow water layers (< 20 m from the surface). The fact that the higher activity levels only occur for males during spawning suggests that it reflects higher levels of agonistic interactions between males linked to mating competition. In contrast, heightened activity levels associated with courtship behavior should arguably lead to a concurrent increase in female activity levels.

### 4.1 Net‐Pen Study

Animals often show different behavior in the laboratory than in their natural environment. This could directly relate to the stress of captivity, or it may be caused by increased density in the laboratory compared to the field. On the one hand, agonistic male—male interactions may increase in higher densities simply because a male encounters more males (de Jong et al. 2012); on the other hand, competitive behavior could break down in the presence of too many competitors (Fenderson and Carpenter 1971; Weir et al. 2011). For spawning cod, the present results clearly indicate the former. Frequent agonistic interactions were indeed observed directly in the net‐pen study (Rugetveit 2024). When fish identity and hence sex could be identified, this involved male fish. This aligns with the observation that male activity levels as detected by the accelerometer tags were very high and much exceeding that of females in the net‐pens.

Agonistic interaction is widespread in animals and known to induce stress, especially during crowding and reproduction (MacLeod et al. 2023). Stress during breeding is also often associated with competition and crowding (MacLeod et al. 2023) and, as is the case for cod (Rose 1993; Nordeide and Folstad 2000), breeding aggregations often represent a peak in density in animals usually living in less dense groups. Dean et al. (2014) noted that male cod on a spawning ground off Massachusetts showed home ranges with little overlap and questioned whether the high levels of aggressive interactions typically observed in laboratory studies (Brawn 1961b; Hutchings et al. 1999; Skjæraasen, Meager, and Hutchings 2010; Skjæraasen, Meager, Karlsen, et al. 2010) would be representative of how mating plays out in the wild. Our findings clearly support this notion. Agonistic interactions are costly in terms of time, energy, and risk of injury (Forkman and Haskell 2004; Beaulieu et al. 2014), but stable dominance hierarchies reducing such behaviors may be less likely to develop when fish are held at high densities (Sverdrup et al. 2011). Agonistic interaction levels can be reduced via local dispersal (Westcott 1997), subordinate avoidance of dominant male territories (Morales et al. 2014), and habitat complexity, allowing subordinates to avoid such interactions by seeking shelter in habitat refugia (Ruberto et al. 2024). The confined net‐pen situation minimizes the opportunity for all these modes of conflict de‐escalation compared to the natural situation in the field.

### 4.2 Field Study

**FIGURE 5:**
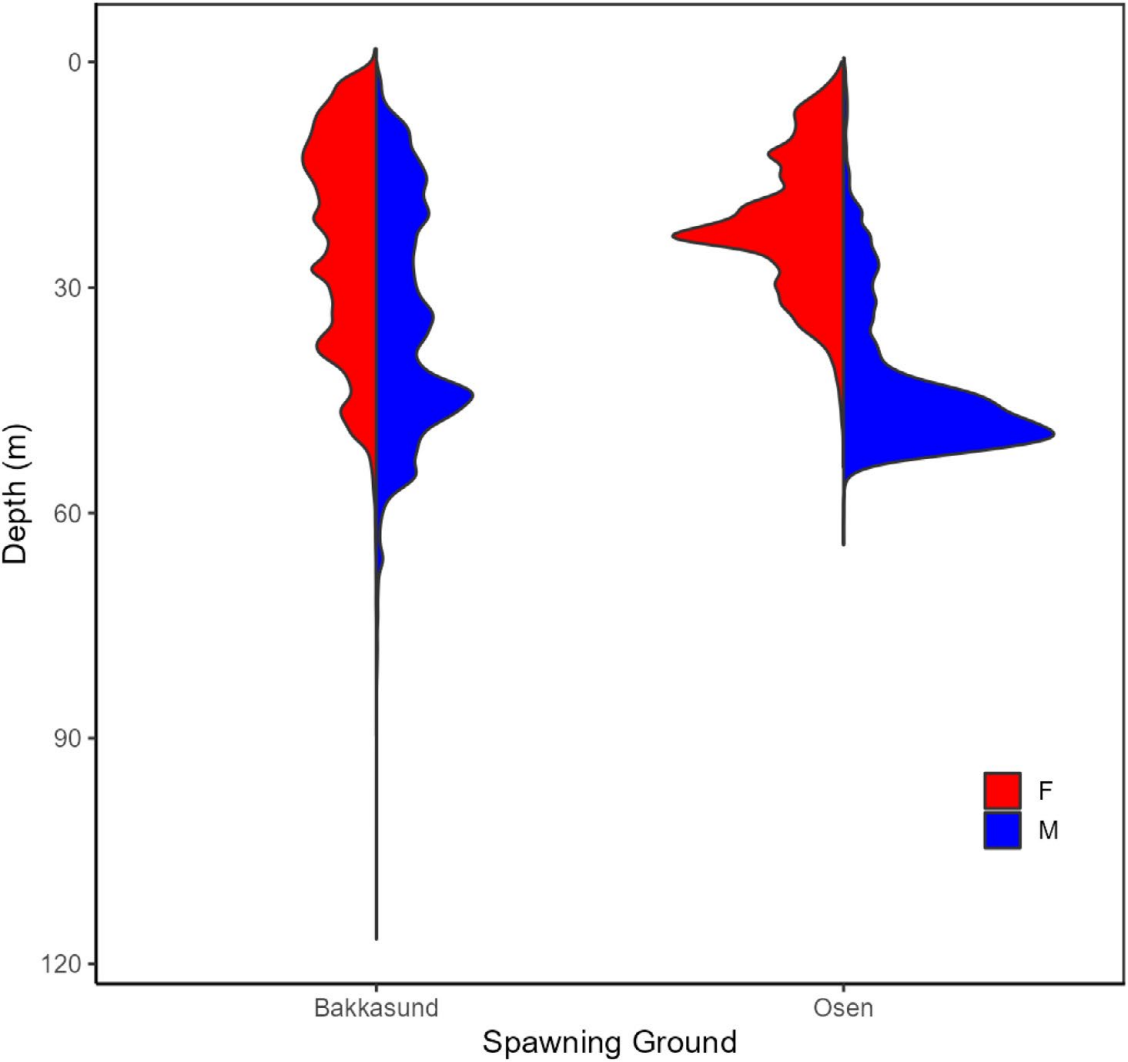
Density distribution of depths during the spawning period for the Bakkasund and Osen spawning grounds for males (M) and females (F).

Lekking is a relatively rare but extensively studied phenomenon shedding light on sexual selection and mating strategies (Balmford 1991; Kokko 1997; Parrish and Edelstein‐Keshet 1999). In leks, males typically aggregate, likely to increase attraction for females to the area, and then defend territories devoid of any resources within the lek, directly competing with one another for access to mates (Rathore et al. 2023). Even though male activity levels were much reduced on the natural spawning grounds compared to the net‐pen situation, males still showed significantly higher activity levels than females on both study grounds during the spawning period. No such difference in activity levels was seen at other times of the year. This is consistent with male–male agonistic interactions being associated with spawning and the description of the cod mating system as a lek (Nordeide and Folstad 2000). Thus, the present results, and notably the difference in both overall activity levels and the proportion of high activity values (> 2 m/s^2^) between males and females at depths deeper than 20 m, agree with this description. Male cod overall stay deeper than females during the spawning period along the Norwegian coast (Meager et al. 2009; Barth et al. 2019; Skjæraasen et al. 2024). The present study fish conformed to this pattern, with male depths clearly deeper overall than females (Figure 5 and Figure S3) at both spawning grounds. Female cod will perform periodic descents towards male depths, with these descents putatively associated with courtship and mate choice, egg release, and spawning (Skjæraasen et al. 2024). Following the lek hypothesis, male agonistic interactions toward other males and thereby male activity levels are expected to be more elevated at depth than those of females, which is indeed what we observe here.

The present study also highlights the need for future studies to advance understanding of the cod mating system. Here, we looked at the overall activity levels of fish and the proportion of high activity values for both sexes. Males may be acting particularly aggressively toward other males in core areas or defend a territory where they spend most of their time, as is commonly observed in lekking species (Rathore et al. 2023). This aspect could be examined further by individual home range analyses in combination with the identification of possible spatial and temporal patterns in activity levels, focusing on where and when the highest values occur. Such studies would be especially useful on spawning grounds where a substantial proportion of spawners can be tagged. Similarly, the female periodic descents toward male depths (Skjæraasen et al. 2024) may also be associated with somewhat elevated activity levels for females at depth, a question also worth further exploration.

A large number of fish were tagged and provided data for the present study, and the difference in activity levels was clear and pronounced during the spawning period and absent otherwise. We thus feel confident that our results reflect real differences in activity levels between the sexes on these spawning grounds and that these differences are related to the cod mating system. This does not necessarily mean that the same sex‐dependent patterns of activity are ubiquitous for spawning cod, as population differences in mating strategies may exist (Dean et al. 2014). Second, we believe that the most parsimonious explanation for our results is antagonistic interactions between males. However, this is inferred from the observed activity levels and not from direct observation in the field. There are other behaviors, such as feeding, that would cause high activity levels (Nakamura et al. 2011; Watanabe et al. 2019), and this could influence our results somewhat, but we do not believe it is likely that such behaviors should cause the sex difference observed only during the spawning period and in deeper waters as observed here.

## 5 Conclusions

In conclusion, this study provides evidence that levels of male Atlantic cod agonistic behavior in close confinement, such as laboratory and net‐pen studies, likely are elevated compared to the natural situation, but that male—male agonistic interactions also occur on natural spawning grounds. Along the Norwegian coast, male cod occupy deeper waters than females during spawning. This is likely associated with territorial behavior and male–male agonistic interactions, causing heightened male activity levels at depth in line with the lek description of the cod mating system. We hypothesize that the same may apply to cod populations elsewhere where such depth segregations have been observed.

## Author Contributions

**J. E. Skjæraasen:** conceptualization (lead), data curation (equal), formal analysis (lead), funding acquisition (supporting), investigation (equal), methodology (equal), visualization (lead), writing – original draft (lead), writing – review and editing (lead). **P. Arechavala‐Lopez:** conceptualization (supporting), formal analysis (supporting), methodology (supporting), writing – original draft (supporting), writing – review and editing (supporting). **Ø. Karlsen:** conceptualization (supporting), funding acquisition (supporting), project administration (supporting), writing – original draft (supporting), writing – review and editing (supporting). **E. M. Olsen:** conceptualization (supporting), funding acquisition (supporting), investigation (supporting), writing – original draft (supporting), writing – review and editing (supporting). **J. J. Meager:** conceptualization (supporting), investigation (supporting), writing – original draft (supporting), writing – review and editing (supporting). **D. Nyqvist:** conceptualization (supporting), investigation (supporting), methodology (supporting), writing – original draft (supporting), writing – review and editing (supporting). **L. S. Doksæter:** conceptualization (supporting), funding acquisition (lead), investigation (supporting), methodology (supporting), project administration (lead), writing – original draft (supporting), writing – review and editing (supporting). **K. McQueen:** conceptualization (supporting), data curation (supporting), investigation (supporting), methodology (lead), project administration (supporting), writing – original draft (supporting), writing – review and editing (supporting). **K. de Jong:** conceptualization (supporting), investigation (supporting), methodology (supporting), project administration (supporting), writing – original draft (supporting), writing – review and editing (supporting). **K. H. Rugtveit:** investigation (supporting), writing – original draft (supporting), writing – review and editing (supporting). **I. A. Catalan:** conceptualization (supporting), formal analysis (supporting), writing – original draft (supporting), writing – review and editing (supporting). **Ø. Langangen:** conceptualization (supporting), formal analysis (equal), visualization (supporting), writing – original draft (supporting), writing – review and editing (supporting).

## Funding

This work was supported by Havforskningsinstituttet, 15338, 16067, Norges Forskningsråd, 280367.

## Conflicts of Interest

The authors declare no conflicts of interest.

## Supporting information


**Data S1:** Supporting Information.

## Data Availability

Data and the source script are available for download from the Norwegian Marine Data Centre (NDMC) (https://doi.org/10.21335/NMDC‐1068069161).

## References

[ece373316-bib-0001] Balmford, A. 1991. “Mate Choice on Leks.” Trends in Ecology & Evolution 6: 87–92. 21232432 10.1016/0169-5347(91)90181-V

[ece373316-bib-0002] Barth, J. M. , D. Villegas‐Ríos , C. Freitas , et al. 2019. “Disentangling Structural Genomic and Behavioural Barriers in a Sea of Connectivity.” Molecular Ecology 28: 1394–1411. 30633410 10.1111/mec.15010 PMC6518941

[ece373316-bib-0003] Barton, K. 2020. “MuMIn: Multi‐Model Inference.” R package version1.43.17. https://CRAN.R‐project.org/package=MuMIn.

[ece373316-bib-0004] Bates, D. , M. Maechler , B. M. Bolker , and S. C. Walker . 2015. “Fitting Linear Mixed‐Effects Models Using lme4.” Journal of Statistical Software 67: 1–48.

[ece373316-bib-0005] Beaulieu, M. , S. Mboumba , E. Willaume , P. M. Kappeler , and M. J. E. Charpentier . 2014. “The Oxidative Cost of Unstable Social Dominance.” Journal of Experimental Biology 217: 2629–2632. 24902748 10.1242/jeb.104851

[ece373316-bib-0006] Bekkevold, D. 2006. “Male Size Composition Affects Male Reproductive Variance in Atlantic Cod *Gadus morhua* L. Spawning Aggregations.” Journal of Fish Biology 69: 945–950.

[ece373316-bib-0007] Berglund, A. 1997. “Mating Systems and Sex Allocation.” In Behavioural Ecology of Teleost Fishes, 237–265. Oxford University Press.

[ece373316-bib-0008] Brawn, V. M. 1961a. “Aggressive Behaviour in the Cod (*Gadus callarias* L.).” Behaviour 18: 107–147.

[ece373316-bib-0009] Brawn, V. M. 1961b. “Reproductive Behaviour of the Cod (Gadus Callarias L.).” Behaviour 18: 177–198.

[ece373316-bib-0010] Broell, F. , C. Burnell , and C. T. Taggart . 2016. “Measuring Abnormal Movements in Free‐Swimming Fish With Accelerometers: Implications for Quantifying Tag and Parasite Load.” Journal of Experimental Biology 219: 695–705. 26747901 10.1242/jeb.133033

[ece373316-bib-0011] Bro‐Jorgensen, J. 2002. “Overt Female Mate Competition and Preference for Central Males in a Lekking Antelope.” Proceedings of the National Academy of Sciences of the United States of America 99: 9290–9293. 12089329 10.1073/pnas.142125899 PMC123133

[ece373316-bib-0012] Brooks, M. E. , K. Kristensen , K. J. Van Benthem , et al. 2017. “glmmTMB Balances Speed and Flexibility Among Packages for Zero‐Inflated Generalized Linear Mixed Modeling.” R Journal 9: 378–400.

[ece373316-bib-0013] de Jong, K. , E. Forsgren , H. Sandvik , and T. Amundsen . 2012. “Measuring Mating Competition Correctly: Available Evidence Supports Operational Sex Ratio Theory.” Behavioral Ecology 23: 1170–1177.

[ece373316-bib-0014] Dean, M. J. , W. S. Hoffman , D. R. Zemeckis , and M. P. Armstrong . 2014. “Fine‐Scale Diel and Gender‐Based Patterns in Behaviour of Atlantic Cod ( *Gadus morhua* ) on a Spawning Ground in the Western Gulf of Maine.” ICES Journal of Marine Science 71: 1474–1489.

[ece373316-bib-0015] Douma, J. C. , and J. T. Weedon . 2019. “Analysing Continuous Proportions in Ecology and Evolution: A Practical Introduction to Beta and Dirichlet Regression.” Methods in Ecology and Evolution 10: 1412–1430.

[ece373316-bib-0016] Fenderson, O. C. , and M. R. Carpenter . 1971. “Effects of Crowding on the Behaviour of Juvenile Hatchery and Wild Landlocked Atlantic Salmon ( *Salmo salar* L.).” Animal Behaviour 19: 439–447.

[ece373316-bib-0017] Forkman, B. , and M. J. Haskell . 2004. “The Maintenance of Stable Dominance Hierarchies and the Pattern of Aggression: Support for the Suppression Hypothesis.” Ethology 110: 737–744.

[ece373316-bib-0018] Hawkins, A. D. , and K. J. Rasmussen . 1978. “The Calls of Gadoid Fish.” Journal of the Marine Biological Association of the United Kingdom 58: 891–911.

[ece373316-bib-0019] Helfman, G. S. , B. B. Collette , D. E. Facey , and B. W. Bowen . 2009. The Diversity of Fishes: Biology, Evolution, and Ecology. John Wiley & Sons.

[ece373316-bib-0020] Howes, G. J. 1991. “Biogeography of Gadoid Fishes.” Journal of Biogeography 18: 595–622.

[ece373316-bib-0021] Hurvich, C. M. , and C.‐L. Tsai . 1989. “Regression and Time Series Model Selection in Small Samples.” Biometrika 76: 297–307.

[ece373316-bib-0022] Hutchings, J. A. , T. D. Bishop , and C. R. McGregor‐Shaw . 1999. “Spawning Behaviour of Atlantic Cod, *Gadus morhua* : Evidence of Mate Competition and Mate Choice in a Broadcast Spawner.” Canadian Journal of Fisheries and Aquatic Sciences 56: 97–104.

[ece373316-bib-0023] Hutchings, J. A. , and R. W. Rangeley . 2011. “Correlates of Recovery for Canadian Atlantic Cod (*Gadus morhua*).” Canadian Journal of Zoology 89: 386–400.

[ece373316-bib-0024] Karlsen, Ø. , and J. C. Holm . 1994. “Ultrasonography, a Noninvasive Method for Sex Determination in Cod (*Gadus morhua*).” Journal of Fish Biology 44: 965–971.

[ece373316-bib-0025] Kokko, H. 1997. “The Lekking Game: Can Female Choice Explain Aggregated Male Displays?” Journal of Theoretical Biology 187: 57–64.

[ece373316-bib-0026] Lennox, R. J. , S. H. Eldoy , L. S. Dahlmo , J. K. Matley , and K. W. Vollset . 2023. “Acoustic Accelerometer Transmitters and Their Growing Relevance to Aquatic Science.” Movement Ecology 11. 10.1186/s40462-023-00403-3. PMC10375738 37501158

[ece373316-bib-0027] MacLeod, K. J. , S. English , S. K. Ruuskanen , and B. Taborsky . 2023. “Stress in the Social Context: A Behavioural and Eco‐Evolutionary Perspective.” Journal of Experimental Biology 226. 10.1242/jeb.245829. PMC10445731 37529973

[ece373316-bib-0028] Martinez‐Garcia, L. , G. Ferrari , A. K. Hufthammer , et al. 2022. “Ancient DNA Reveals a Southern Presence of the Northeast Arctic Cod During the Holocene.” Biology Letters 18. 10.1098/rsbl.2022.0021. PMC9065953 35506242

[ece373316-bib-0029] Marttila, M. 2024. “censlm: Censored Linear Models.” R package version 0.0.0.9001. https://github.com/mikmart/censlm.

[ece373316-bib-0030] McQueen, K. , J. J. Meager , D. Nyqvist , et al. 2022. “Spawning Atlantic Cod ( *Gadus morhua* L.) Exposed to Noise From Seismic Airguns Do Not Abandon Their Spawning Site.” ICES Journal of Marine Science 79: 2697–2708.

[ece373316-bib-0031] McQueen, K. , L. D. Sivle , T. N. Forland , et al. 2024. “Continuous Sound From a Marine Vibrator Causes Behavioural Responses of Free‐Ranging, Spawning Atlantic Cod ( *Gadus morhua* ).” Environmental Pollution 344: 123322. 38211875 10.1016/j.envpol.2024.123322

[ece373316-bib-0032] McQueen, K. , L. D. Sivle , B. Khodabandeloo , J. E. Skjæraasen , E. M. Olsen , and Ø. Karlsen . 2025. “Free‐Ranging Atlantic Cod Did Not Change Their Behaviour in Response to a Sparker Seismic Sound Source.” Marine Environmental Research 210: 107254. 40482351 10.1016/j.marenvres.2025.107254

[ece373316-bib-0033] McQueen, K. , J. E. Skjæraasen , D. Nyqvist , et al. 2023. “Behavioural Responses of Wild, Spawning Atlantic Cod ( *Gadus morhua* L.) to Seismic Airgun Exposure.” ICES Journal of Marine Science 80: 1052–1065.

[ece373316-bib-0034] Meager, J. J. , J. E. Skjæraasen , A. Ferno , et al. 2009. “Vertical Dynamics and Reproductive Behaviour of Farmed and Wild Atlantic Cod *Gadus morhua* .” Marine Ecology Progress Series 389: 233–243.

[ece373316-bib-0035] Morales, M. B. , F. Casas , E. García de la Morena , et al. 2014. “Density Dependence and Habitat Quality Modulate the Intensity of Display Territory Defence in an Exploded Lekking Species.” Behavioral Ecology and Sociobiology 68: 1493–1504.

[ece373316-bib-0036] Morgan, M. J. , and E. A. Trippel . 1996. “Skewed Sex Ratios in Spawning Shoals of Atlantic Cod ( *Gadus morhua* ).” ICES Journal of Marine Science 53: 820–826.

[ece373316-bib-0037] Morgan, M. J. , C. E. Wilson , and L. W. Crim . 1999. “The Effect of Stress on Reproduction in Atlantic Cod.” Journal of Fish Biology 54: 477–488.

[ece373316-bib-0038] Munoz, R. C. , B. J. Zgliczynski , B. Z. Teer , and J. L. Laughlin . 2014. “Spawning Aggregation Behavior and Reproductive Ecology of the Giant Bumphead Parrotfish, Bolbometopon Muricatum, in a Remote Marine Reserve.” PeerJ 2: e681. 25469322 10.7717/peerj.681 PMC4250069

[ece373316-bib-0039] Nakamura, I. , Y. Y. Watanabe , Y. P. Papastamatiou , K. Sato , and C. G. Meyer . 2011. “Yo‐Yo Vertical Movements Suggest a Foraging Strategy for Tiger Sharks *Galeocerdo cuvier* .” Marine Ecology Progress Series 424: 237–246.

[ece373316-bib-0040] Nelson, J. , P. Gotwalt , S. Reidy , and D. Webber . 2002. “Beyond Ucrit: Matching Swimming Performance Tests to the Physiological Ecology of the Animal, Including a New Fish ‘Drag Strip’.” Comparative Biochemistry and Physiology Part A: Molecular & Integrative Physiology 133: 289–302. 10.1016/s1095-6433(02)00161-7 12208301

[ece373316-bib-0041] Nordeide, J. T. , and I. Folstad . 2000. “Is Cod Lekking or a Promiscuous Group Spawner?” Fish and Fisheries 1: 90–93.

[ece373316-bib-0042] Olsen, E. M. , Ø. Karlsen , and J. E. Skjæraasen . 2023. “Large Females Connect Atlantic Cod Spawning Sites.” Science 382: 1181–1184. 38060630 10.1126/science.adi1826

[ece373316-bib-0043] Parrish, J. K. , and L. Edelstein‐Keshet . 1999. “Complexity, Pattern, and Evolutionary Trade‐Offs in Animal Aggregation.” Science 284: 99–101. 10102827 10.1126/science.284.5411.99

[ece373316-bib-0068] R Core Team . 2023. “_R: A Language and Environment for Statistical Computing_.” R Foundation for Statistical Computing, Vienna, Austria.

[ece373316-bib-0044] Rathore, A. , K. Isvaran , and V. Guttal . 2023. “Lekking as Collective Behaviour.” Philosophical Transactions of the Royal Society B 378: 20220066. 10.1098/rstb.2022.0066 PMC9939265 36802778

[ece373316-bib-0045] Rose, G. A. 1993. “Cod Spawning on a Migration Highway in the North‐West Atlantic.” Nature 366: 458–461.

[ece373316-bib-0046] Rowe, S. , and J. A. Hutchings . 2006. “Sound Production by Atlantic Cod During Spawning.” Transactions of the American Fisheries Society 135: 529–538.

[ece373316-bib-0047] Rowe, S. , J. A. Hutchings , J. E. Skjæraasen , and L. Bezanson . 2008. “Morphological and Behavioural Correlates of Reproductive Success in Atlantic Cod *Gadus morhua* .” Marine Ecology‐Progress Series 354: 257–265.

[ece373316-bib-0048] Ruberto, T. , W. T. Swaney , and A. R. Reddon . 2024. “Submissive Behavior Is Affected by Territory Structure in a Social Fish.” Current Zoology 70: 803–809. 39678816 10.1093/cz/zoae014 PMC11634681

[ece373316-bib-0069] Rugetveit, K. H. 2024. “The Effect of Seismic Shooting on the Spawning Behaviour of Cod (*Gadus morhua*).” Master thesis, University of Bergen – Department of Biological Sciences, Norway.

[ece373316-bib-0049] Shorey, L. 2002. “Mating Success on White‐Bearded Manakin (*Manacus manacus*) Leks: Male Characteristics and Relatedness.” Behavioral Ecology and Sociobiology 52: 451–457.

[ece373316-bib-0050] Skjæraasen, J. E. , J. J. Meager , and M. Heino . 2012. “Secondary Sexual Characteristics in Codfishes (Gadidae) in Relation to Sound Production, Habitat Use, and Social Behaviour.” Marine Biology Research 8: 201–209.

[ece373316-bib-0051] Skjæraasen, J. E. , J. J. Meager , and J. A. Hutchings . 2010. “A Cost of Reproduction in Male Atlantic Cod ( *Gadus morhua* ).” Canadian Journal of Zoology 88: 595–600.

[ece373316-bib-0052] Skjæraasen, J. E. , J. J. Meager , Ø. Karlsen , et al. 2010. “Mating Competition Between Farmed and Wild Cod *Gadus morhua* .” Marine Ecology‐Progress Series 412: 247–258.

[ece373316-bib-0053] Skjæraasen, J. E. , E. M. Olsen , K. McQueen , et al. 2024. “Sex‐Specific Vertical Movements of Spawning Atlantic Cod in Coastal Habitats Inferred From Acoustic Telemetry.” Scientific Reports 14: 23242. 39369150 10.1038/s41598-024-74896-2 PMC11455899

[ece373316-bib-0054] Sloman, K. A. , and J. D. Armstrong . 2002. “Physiological Effects of Dominance Hierarchies: Laboratory Artefacts or Natural Phenomena?” Journal of Fish Biology 61: 1–23.

[ece373316-bib-0055] Small, J. , S. Cotton , K. Fowler , and A. Pomiankowski . 2009. “Male Eyespan and Resource Ownership Affect Contest Outcome in the Stalk‐Eyed Fly, Teleopsis Dalmanni.” Animal Behaviour 78: 1213–1220.

[ece373316-bib-0056] Smith, C. , and R. J. Wootton . 2016. “The Remarkable Reproductive Diversity of Teleost Fishes.” Fish and Fisheries 17: 1208–1215.

[ece373316-bib-0057] Stockley, P. , M. J. G. Gage , G. A. Parker , and A. P. Moller . 1997. “Sperm Competition in Fishes: The Evolution of Testis Size and Ejaculate Characteristics.” American Naturalist 149: 933–954. 10.1086/286031 18811256

[ece373316-bib-0058] Sverdrup, G. K. , J. J. Meager , A. Fernö , et al. 2011. “Territorial and Agonistic Interactions Between Farmed and Wild Cod ( *Gadus morhua* ).” Aquaculture Research 42: 1539–1548.

[ece373316-bib-0059] Thorsen, A. , and O. S. Kjesbu . 2001. “A Rapid Method for Estimation of Oocyte Size and Potential Fecundity in Atlantic Cod Using a Computer‐Aided Particle Analysis System.” Journal of Sea Research 46: 295–308.

[ece373316-bib-0067] van der Knaap, I. , J. Reubens , L. Thomas , et al. 2021. “Effects of a Seismic Survey on Movement of Free‐Ranging Atlantic Cod.” Current Biology 31, no. 7: 1555–1562.e4. 10.1016/j.cub.2021.01.050. 33567289

[ece373316-bib-0060] Videler, J. 1981. Swimming Movements, Body Structure and Propulsion in Cod *Gadus morhua* . Symposia of the Zoological Society.

[ece373316-bib-0061] Villegas‐Ríos, D. , C. Freitas , E. Moland , S. H. Thorbjørnsen , and E. M. Olsen . 2020. “Inferring Individual Fate From Aquatic Acoustic Telemetry Data.” Methods in Ecology and Evolution 11: 1186–1198.

[ece373316-bib-0062] Watanabe, Y. Y. , N. L. Payne , J. M. Semmens , A. Fox , and C. Huveneers . 2019. “Hunting Behaviour of White Sharks Recorded by Animal‐Borne Accelerometers and Cameras.” Marine Ecology Progress Series 621: 221–227.

[ece373316-bib-0063] Weir, L. K. , J. W. Grant , and J. A. Hutchings . 2011. “The Influence of Operational Sex Ratio on the Intensity of Competition for Mates.” American Naturalist 177: 167–176. 10.1086/657918 21460553

[ece373316-bib-0064] Westcott, D. A. 1997. “Neighbours, Strangers and Male‐Male Aggression as a Determinant of Lek Size.” Behavioral Ecology and Sociobiology 40: 235–242.

[ece373316-bib-0065] Wickham, H. , M. Averick , J. Bryan , et al. 2019. “Welcome to the Tidyverse.” Journal of Open Source Software 4: 1686.

[ece373316-bib-0066] Windle, M. J. S. , and G. A. Rose . 2007. “Do Cod Form Spawning Leks? Evidence From a Newfoundland Spawning Ground.” Marine Biology 150: 671–680.

