# Supplementary material for: Male Agonistic Behavior on Atlantic Cod Spawning Grounds
Source: Ecol Evol. 2026 Mar 27;16(4):e73316. doi: 10.1002/ece3.73316 (PMC13107262; doi:10.1002/ece3.73316)

**Supplementary material - Skjæraasen et al**

**Supplementary Table 1.** Summary of commonly observed cod mating and spawning behaviours. Table is modified from (Skjæraasen *et al.* 2010). (Brawn 1961; Hutchings, Bishop & McGregor-Shaw 1999). Behaviours in bold font and italics assumed to cause high activity levels detectable as high values in the accelerometer tags.

| **Male – Female Courtship** | **Source** | **Description** |
| --- | --- | --- |
| Ventral mount | Brawn (1961) | A male cod slides down the side of a female, ending up stomach to stomach with gonadal pores close together. This is the behaviour commonly associated with spawning. |
| Paired swim | Brawn (1961) | A male swims towards a female and follows her motion. Typically resulting in a circling pattern and often ending in a ventral mount. |
| Circling | Hutchings et al. (1999) | A female cod rests on the bottom, while a male swims in circles above. |
| Lateral display | Nilsson (2004) | A cod approaches another cod and freezes in mid-water, flexing its pectoral and pelvic fins. |
| **Male – male agonistic interactions** |  |  |
| ***Approach*** | Hutchings et al. (1999) | A cod swims rapidly towards another cod, stopping before contact has been made. Typically causing the recipient to flee. |
| ***Chase*** | Brawn (1961) | A cod swims rapidly towards another cod. Commonly invoking a flee response in the recipient with the aggressor sometimes continuing to chase the other cod after it starts to flee. |
| ***Prod*** | Hutchings et al. (1999) | A cod swims into the side of another cod, making contact with its snout. Typically causing the recipient to flee. |
| ***Nip/bite*** | Hutchings et al. (1999) | A cod bites or attempts to bite another cod. Typically causing the recipient to flee. |

**Supplementary Table 2** Data periods omitted from the net-pen study and the field study in Bakkasund.

| **Study** | **Exposure Start** | **Exposure End** |
| --- | --- | --- |
| Net – pen | 01.04.2019 | 06.04.2019 |
| Net – pen | 09.03.2020 | 11.03.2020 |
| Bakkasund | 10.02.2020 | 14.02.2020 |
| Bakkasund | 15.02.2021 | 20.02.2021 |
| Bakkasund | 13.02.2022 | 17.02.2022 |
| Bakkasund | 28.02.2024 | 16.06.2023 |
| Bakkasund | 30.06.2025 | 01.03.2024 |
| Bakkasund | 27.05.2023 | 03.07.2025 |

**Supplementary Table 3.** Activity levels, response variable, during the feeding period.

|  | **Activity levels - Bakkasund** | | | **Activity levels -Osen** | | |
| --- | --- | --- | --- | --- | --- | --- |
| *Predictors* | *Estimates* | *CI* | *p* | *Estimates* | *CI* | *p* |
| (Intercept) | 0.91 | 0.80 – 1.02 | **<0.001** | -0.22 | -1.78 – 1.34 | 0.770 |
| Length |  |  |  | 0.00 | -0.00 – 0.00 | 0.276 |
| **Random Effects** | | | | | | |
| σ^2^ | 0.39 | | |  | | |
| τ_00_ | 0.01 _Year_ | | |  | | |
| ICC | 0.03 | | |  | | |
| N | 6 _Year_ | | |  | | |
| Observations | 59 | | | 19 | | |
| Marginal R^2^ / Conditional R^2^ | 0.000 / 0.028 | | | 0.069 / 0.015 | | |

Right - sensored data deliver a maximum value and any value at or above this value is still delivered as this maximum value, typically resulting in a peak in the distribution of values at this maximum value. This is very much the case for our accelerometer data (e.g. Fig. S2 top panel). One way to be able to deal statistically with such data is to impute or assign new values to any data points at this maximum value. We used the censlm package of R (Marttila 2024) to do this. The method used first log – transforms the data and then uses a decay function, i.e. a linear fit, to give new values to the censored data. We applied this method to all activity level data with accelerometer values above 1 (to avoid the large number of small values affecting the imputed values) to produce the datasets used for analyses (e.g. Fig S2 bottom panel). To test if the threshold in the accelerometer may affect our results, we also compared models with and without imputation of the data above the threshold acceleration values. This test indicated that this was not the case.

Brawn, V.M. (1961) Reproductive behaviour of the cod (Gadus callarias L.). *Behaviour,* **18,** 177–198.

Hutchings, J.A., Bishop, T.D. & McGregor-Shaw, C.R. (1999) Spawning behaviour of Atlantic cod, *Gadus morhua*: evidence of mate competition and mate choice in a broadcast spawner. *Canadian Journal of Fisheries and Aquatic Sciences,* **56,** 97-104.

Marttila, M. (2024) _censlm: Censored Linear Models_. R package version 0.0.0.9001, <<https://github.com/mikmart/censlm>>.

Skjæraasen, J.E., Meager, J.J., Karlsen, Ø., Mayer, I., Dahle, G., Rudolfsen, G. & Fernö, A. (2010) Mating competition between farmed and wild cod *Gadus morhua*. *Marine Ecology-Progress Series,* **412,** 247-258.

**Supplementary figure 1.** Schematic of the net-pens used in the net-pen study.


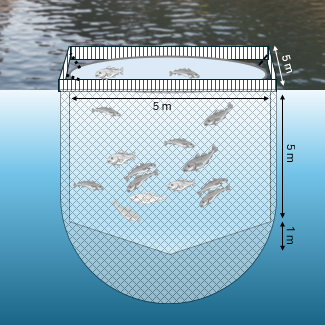


**Supplementary figure 2.** Raw and imputed accelereometer data, i.e. activity levels, data from the net-pen. Top panel is the complete raw dataset before data imputation, and bottom panel is the complete dataset used for the statistical analyses


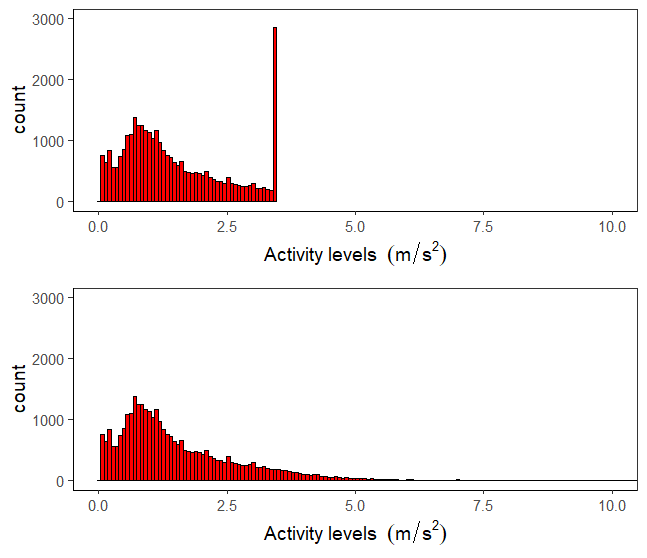


**Supplementary figure 3.** Examples of temporal variation in depth (top panel) and activity data (bottom panel) for one example male (blue) and one female (red) showing two weeks of data during the spawning period of 2024.


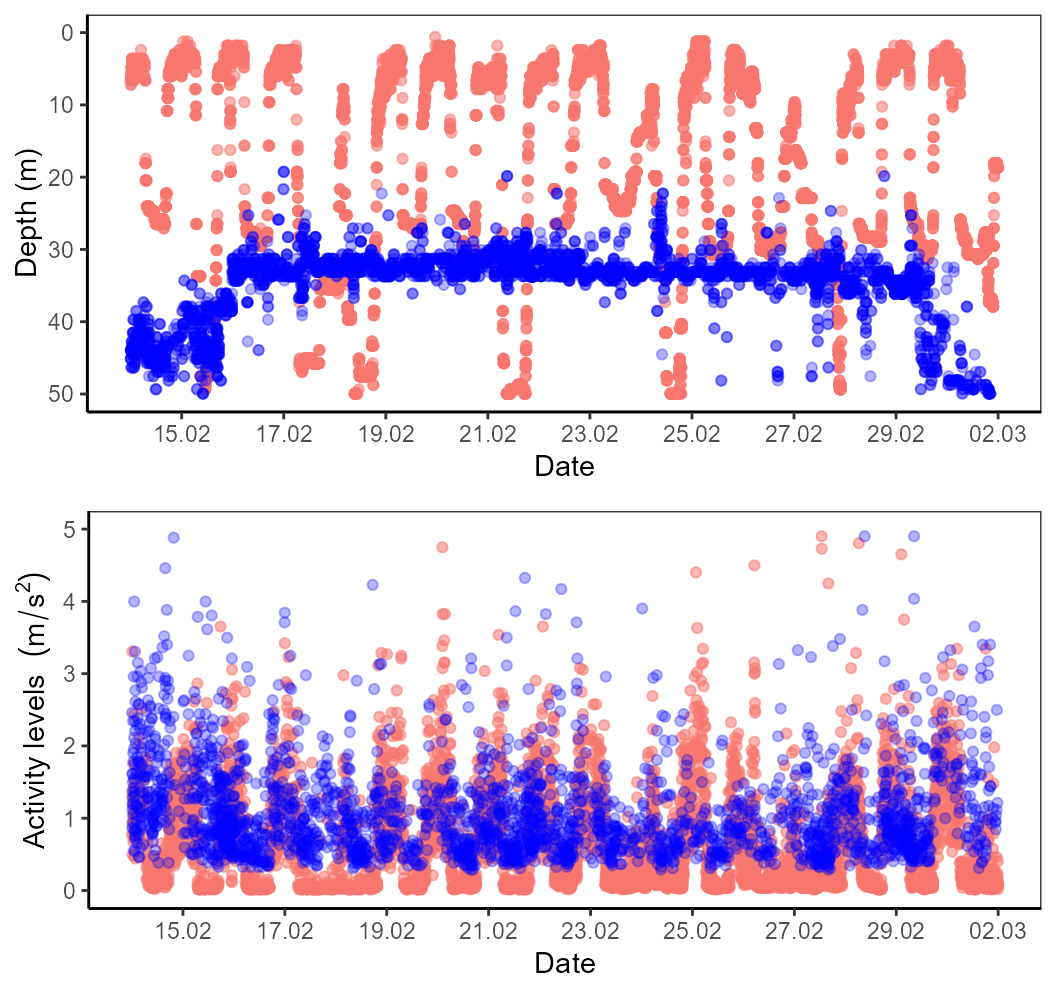

Supplement: Supplementary file 1 — Data S1: Supporting Information. [file ECE3-16-e73316-s001.docx]
